# Severe Unilateral Iliopsoas Atrophy After Dysplastic Hip Arthroplasty Leading to Recurrent L4/L5 Disc Degeneration: A Report of a Biomechanical Case

**DOI:** 10.7759/cureus.100290

**Published:** 2025-12-28

**Authors:** Tomasz Sienkiel, Jakub Kalisz, Marcin Gaska, Barbara Jasiewicz

**Affiliations:** 1 Department of Orthopedics, Jagiellonian University Medical College, Krakow, POL; 2 Department of Orthopedics, University Orthopedic and Rehabilitation Hospital, Zakopane, POL

**Keywords:** endoscopic discectomy, hip–spine syndrome, iliopsoas atrophy, lumbar disc degeneration, multifidus atrophy, pelvic imbalance, total hip arthroplasty (tha)

## Abstract

The coordinated function of the hip, pelvis and lumbar spine is essential for maintaining mechanical stability, and the iliopsoas muscle contributes both to primary hip flexion and anterior lumbar stabilisation. Dysfunction of this muscle after dysplastic total hip arthroplasty (THA) may significantly alter load transmission and predispose to lumbar degeneration. We present the case of a 45-year-old male patient with a history of Perthes disease who underwent dysplastic THA complicated by peroneal nerve palsy, persistent gait asymmetry and pelvic imbalance. Several years later, he developed right-sided L4/L5 radiculopathy treated with full-endoscopic transforaminal discectomy, with only temporary improvement. Quantitative MRI demonstrated marked unilateral iliopsoas atrophy (57-85% CSA reduction), mild multifidus asymmetry and gluteus medius/minimus atrophy. A persistent right-sided L4/L5 foraminal/lateral disc protrusion correlated with recurrent symptoms. The combination of severe iliopsoas degeneration, hip abductor insufficiency and compensatory overload of the multifidus created a biomechanically vulnerable environment in which loss of anterior stabilisation increased shear forces at L4/L5, contributing to recurrent disc failure. This case underscores a muscular mechanism within the hip-spine relationship rarely described in the literature and highlights the importance of assessing psoas and gluteal musculature in patients presenting with lumbar symptoms following THA.

## Introduction

The hip, pelvis and lumbar spine form a tightly integrated kinetic chain in which muscle balance is crucial for maintaining segmental stability. Among these muscles, the iliopsoas plays a dual role: it acts as the primary hip flexor while simultaneously functioning as a major anterior stabiliser of the lumbar spine, supplying stiffness to the anterior column and resisting flexion-induced shear forces, particularly at the L4/L5 level [1-3].

Disruption of this stabilising system, whether through weakness of the iliopsoas, degeneration of the multifidus, or hip abductor insufficiency, can lead to altered pelvic mechanics, compensatory trunk lean and increased mechanical load on the lumbar spine. Such patterns are increasingly recognised within the broader concept of the hip-spine syndrome, originally described by Offierski and MacNab [4], and later expanded by contemporary biomechanical and radiological work.

Total hip arthroplasty (THA), especially in patients with underlying hip dysplasia or childhood disorders such as Perthes disease, may significantly alter pelvic alignment and muscular function. Limb-length changes, tendon tension modifications, postoperative nerve injury and altered activation patterns can all contribute to muscle imbalance. Several MRI-based studies have demonstrated unilateral atrophy of the iliopsoas or hip abductors following THA, though typically of mild-to-moderate severity [5,6].

Despite this, the downstream mechanical consequences on the lumbar spine remain under-recognised. The iliopsoas is uniquely positioned to influence lumbar shear forces due to its vertebral attachments [1-3], and degeneration of this muscle may compromise the stabilising system described by Panjabi [1], predisposing the L4/L5 segment to microinstability, multifidus overload and eventual disc degeneration.

To date, no published case report has clearly demonstrated a causal sequence in which severe unilateral iliopsoas atrophy following THA leads to pelvic imbalance and symptomatic L4/L5 disc disease, supported by detailed quantitative MRI. We present such a case, illustrating a unique mechanism that may explain recurrent lumbar pathology in selected post-THA patients and highlighting the importance of muscular assessment in this population.

## Case presentation

**Figure 1:**
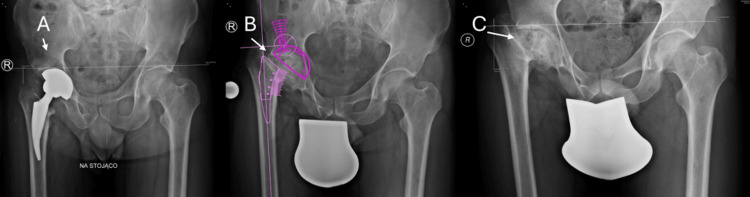
Standing Pelvic Radiographs Demonstrating Post-THA Anatomy and Preoperative Biomechanical Context A: Standing anteroposterior pelvic radiograph demonstrating postoperative alignment after right total hip arthroplasty (THA). B: Post-THA planning radiograph showing limb-length correction and pelvic frontal-plane alignment following right THA. C: Pre-THA radiograph illustrating dysplastic hip morphology and preoperative pelvic imbalance.

A 45-year-old man with a long-standing history of right hip pathology was admitted for evaluation of recurrent right-sided lumbar radiculopathy. He had been diagnosed in childhood with Perthes disease of the right hip, which led to progressive biomechanical deterioration and chronic pain throughout adulthood. In January 2019, he underwent right THA for end-stage degenerative changes. His postoperative course was complicated by neuropraxia of the right common peroneal nerve, confirmed by electromyography, resulting in transient foot-drop. Standing radiographs demonstrated lengthening of the right lower limb by 22 mm, correcting a pre-existing 35 mm shortening and improving frontal-plane pelvic alignment. Following two inpatient rehabilitation programs, he experienced substantial improvement in muscle strength; active dorsiflexion of the right foot and hallux recovered to Lovett grade 4, and overall pelvic stability improved, although a compensatory gait asymmetry persisted due to the nerve deficit (see Figure 1).

In December 2022, the patient developed acute right-sided L4 and partial L5 radiculopathy. Lumbar MRI demonstrated a right-sided foraminal and extraforaminal L4/L5 disc protrusion with significant narrowing of the neural foramen and compression of the exiting L4 root, accompanied by Pfirrmann grade II degeneration but no Modic changes or radiographic signs of instability. These findings correlated with his clinical symptoms. He underwent full-endoscopic transforaminal discectomy, which resulted in complete resolution of radicular pain and functional improvement.

In 2024, he noted a gradual recurrence of low back and right lower-limb pain, radiating to the lateral calf and dorsum of the foot and exacerbated by physical exertion and cold temperatures. Examination revealed global right lower-limb weakness (Lovett 4/5), reduced strength in the tibialis anterior and extensor hallucis longus (Lovett 3-4), diminished sensation over the lateral calf and dorsum of the right foot, a positive straight leg raise at 45°, a compensatory Trendelenburg-type gait, difficulty walking on heels, and visible atrophy of the right gluteus medius and minimus. These findings indicated progressive dysfunction of the hip-pelvis-spine muscular stabilisation system.

**Figure 2:**
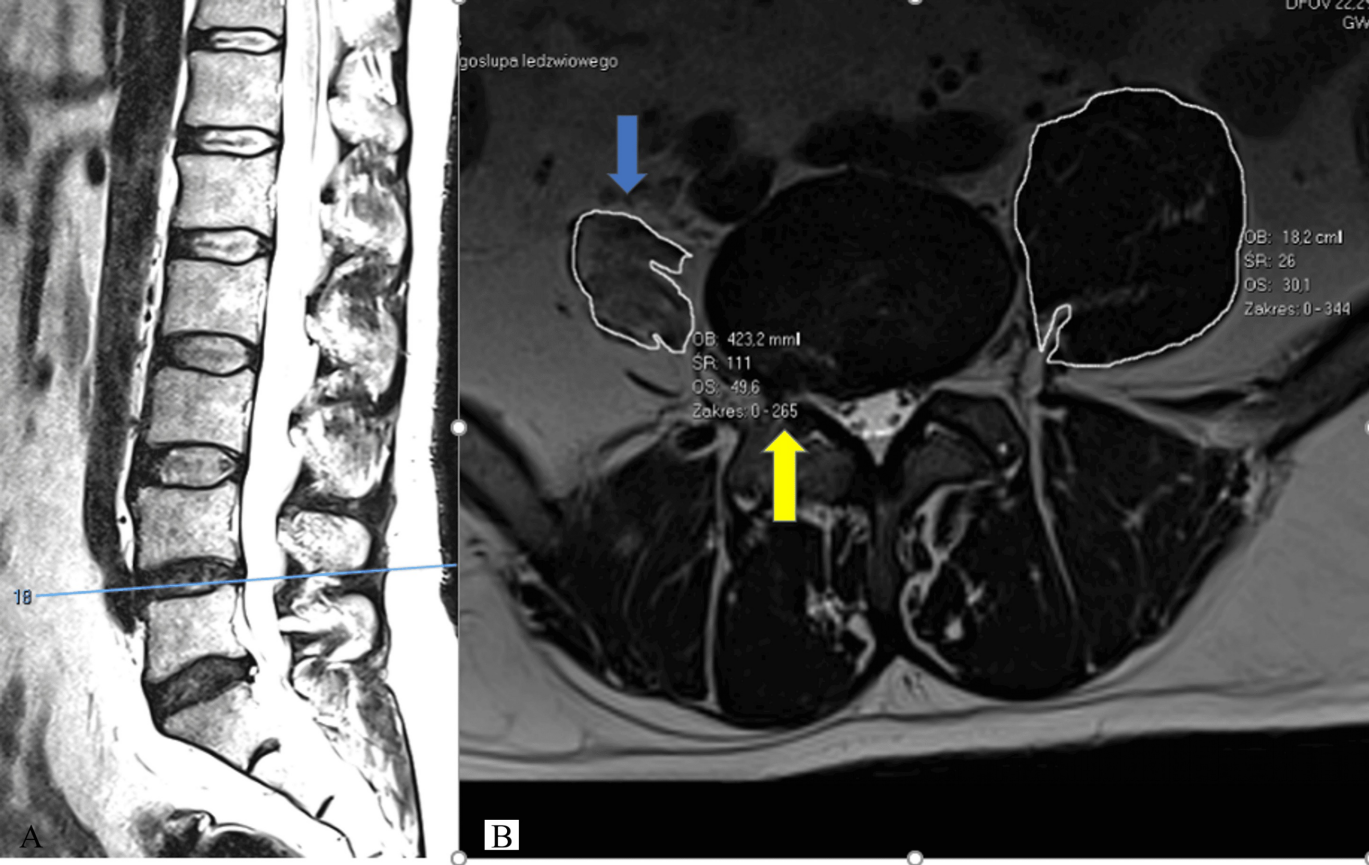
Sagittal and Axial T2-Weighted MRI at the L4/L5 Level A: Sagittal T2-weighted MRI showing the midline levels of L4/L5 and L5/S1, with Pfirrmann grade II degeneration (“black disc”) at both segments. B: Axial T2-weighted MRI at L4/L5. The blue arrow indicates marked unilateral atrophy of the right iliopsoas muscle, while the yellow arrow highlights the right extraforaminal L4/L5 disc protrusion compressing the exiting L4 nerve root.

Follow-up MRI performed in 2024-2025 demonstrated persistent right-sided L4/L5 foraminal and lateral disc protrusion with indentation of the L4 nerve root, without central canal stenosis or segmental instability. Quantitative muscular analysis revealed marked unilateral atrophy of the right iliopsoas with cross-sectional area (CSA) measurements ranging from 157 to 182 mm² on the right compared with 423-780 mm² on the left, corresponding to a 57-85% reduction in muscle volume and fatty infiltration consistent with Goutallier grades 2-3 (see Table 1; Figure 2).

**Table 1:** Quantitative MRI Analysis of Iliopsoas Muscle Atrophy (L2–S1, Right vs. Left) Quantitative MRI analysis demonstrating severe unilateral atrophy of the right iliopsoas muscle across lumbar levels L2–S1. Cross-sectional area (CSA) reduction ranged from 57% to 85% compared with the contralateral side, with fatty infiltration corresponding to Goutallier grades 2–3.

Lumbar Level	Right CSA (mm²)	Left CSA (mm²)	Muscle Loss (%)	Goutallier Grade
L2–L3	182 mm²	423 mm²	≈ 57%	Grade 2
L3–L4	168 mm²	610 mm²	≈ 72%	Grade 2–3
L4–L5	157 mm²	780 mm²	≈ 78%	Grade 3
L5-S1	110 mm²	760 mm²	≈ 85%	Grade 3

Multifidus morphology showed only minimal side-to-side variation at L4/L5 without true atrophy or fatty infiltration, suggesting a functional rather than structural alteration. In contrast, the right gluteus medius and minimus demonstrated visible atrophy, consistent with impaired pelvic stabilisation. Collectively, these findings indicated chronic dysfunction across the anterior, posterior and lateral stabilising systems of the lumbosacral region.

In 2025, due to persistent symptoms, the patient underwent a second full-endoscopic transforaminal discectomy. His postoperative course was favourable, with marked reduction of radicular symptoms and progressive improvement in muscle strength, trunk balance, pelvic girdle stability and gait efficiency during supervised rehabilitation.

## Discussion

The coordinated interaction of the hip, pelvis and lumbar spine is essential for maintaining mechanical stability during upright posture and gait. This interdependence, often referred to as the hip-spine complex, is strongly influenced by the balance of anterior and posterior stabilising muscles. The iliopsoas plays a particularly important role in this system, functioning not only as the primary hip flexor but also as a key contributor to anterior lumbar stabilisation through its attachments to the vertebral bodies and intervertebral discs from T12 to L5 [1-3]. By generating anterior column stiffness and opposing flexion-induced shear at the L4/L5 level, the iliopsoas helps protect the segment most vulnerable to degenerative pathology.

In the present case, quantitative MRI demonstrated profound unilateral atrophy of the right iliopsoas, with a 57-85% reduction in cross-sectional area and fatty infiltration consistent with Goutallier grades 2-3, as shown in Table 1. This degree of atrophy far exceeds the mild, often subclinical changes described in MRI studies following THA [5]. Such severe degeneration suggests long-standing dysfunction, likely eliminating the stabilising contribution of the psoas on the affected side. In accordance with Panjabi’s spinal stability model, the loss of a major muscular stabiliser increases segmental shear, expands the neutral zone and predisposes the spine to microinstability [1].

In addition to psoas degeneration, this patient exhibited visible atrophy of the right gluteus medius and minimus, which are critical frontal-plane stabilisers. Hip abductor insufficiency is well documented after THA, particularly in dysplastic anatomy, and is associated with pelvic drop, limb-length imbalance and compensatory trunk lean [6]. These adaptations shift the centre of mass laterally, increase frontal-plane bending moments and transmit additional mechanical load to the lumbar spine. When abductor weakness is combined with psoas insufficiency, the pelvis becomes unstable in both sagittal and frontal planes, amplifying loading at the L4/L5 segment. This mechanism is supported by the patient's gait abnormalities and by the asymmetric iliopsoas morphology demonstrated on axial MRI (Figure 2).

Although the multifidus showed only minimal side-to-side variation, even subtle changes may reflect compensatory overload when anterior stabilisation is impaired. The multifidus is a primary posterior stabiliser responsible for controlling motion within the neutral zone. Increased demand on this muscle, such as compensating for anterior stabiliser failure, can contribute to fatigue, stiffness or early degenerative change. Systematic reviews have linked multifidus dysfunction to disc pathology and chronic low back pain [7], and in this context, the patient’s mild asymmetry may represent a functional adaptation rather than true atrophy.

The interplay of these deficits, failure of the anterior stabiliser (iliopsoas), weakness of the frontal-plane stabilisers (gluteus medius/minimus) and compensatory reliance on posterior stabilisers (multifidus), created a biomechanical environment highly susceptible to disc failure. Under these conditions, everyday activities such as forward bending, lifting and gait transferred excessive shear forces to the L4/L5 disc, which likely contributed to recurrent herniation despite technically successful endoscopic decompression.

This case reinforces the importance of evaluating hip-spine mechanics when assessing recurrent radiculopathy. Offierski and MacNab originally described the hip-spine syndrome as a clinical entity in which dysfunction in one region influences the other [4]. In this patient, the mechanism was specifically muscular rather than purely structural: postoperative alterations in the hip musculature-exacerbated by dysplastic anatomy-led to secondary destabilisation of the lumbar spine. This expands the classical hip-spine concept by demonstrating a clear, MRI-supported pathway linking THA-related muscle degeneration to symptomatic lumbar disc disease.

Finally, this case highlights that rehabilitation alone may be insufficient to reverse advanced fatty degeneration of the iliopsoas. Although neuromuscular retraining can improve motor control, severe structural atrophy is unlikely to regenerate, leaving patients at risk of ongoing lumbar overload. Early identification of muscle imbalance, particularly with quantitative MRI, may therefore be essential to preventing recurrent lumbar pathology in post-THA patients.

## Conclusions

This case demonstrates that severe unilateral iliopsoas atrophy following dysplastic THA can profoundly disrupt hip-pelvis-spine biomechanics and predispose to segmental lumbar instability. Loss of anterior lumbar stabilisation provided by the iliopsoas, combined with hip abductor insufficiency, led to pelvic imbalance, compensatory multifidus overload and recurrent L4/L5 disc degeneration. Quantitative MRI played a crucial role in identifying these muscular abnormalities and establishing their biomechanical relevance. Clinicians should carefully assess muscular asymmetry and stabiliser dysfunction when evaluating lumbar symptoms in post-THA patients, particularly those with dysplastic anatomy or persistent gait abnormalities, as early recognition of these patterns may help prevent recurrent lumbar pathology.
